# Construct Validity of the Shedler-Westen Assessment Procedure (SWAP-AP)–Emotionally Dysregulated Adolescent Prototype

**DOI:** 10.1192/bjo.2026.11104

**Published:** 2026-06-30

**Authors:** Daniel Napack, Kristina Burns, Emily Kim

**Affiliations:** 1 Northwell Health, Port Jefferson, USA; 2 New York Institute of Technology, Old Westbury, USA

## Abstract

**Aims:**

Assessing personality pathology in adolescence is crucial for early identification of issues that may manifest in adulthood. While adolescence involves typical “storm and stress”, some adolescents exhibit personality pathology beyond normative developmental challenges. Early assessment and intervention are key during this pivotal developmental period. The Shedler–Westen Assessment Procedure–Adolescent Prototypes (SWAP-AP) offers a promising clinical tool for assessing personality functioning. This study aimed to examine the construct validity of the SWAP-AP Emotionally Dysregulated prototype.

**Methods:**

Adolescents and their parents (N=121) from a Long Island, NY suburban adolescent psychiatry outpatient clinic consented to participate in an assessment and treatment outcome study. Participants completed admission packets, including the BeckYouth Inventory (BYI), Inventory of Interpersonal Problems-32 (IIP-32), Youth Outcome Questionnaire (YOQ), and Youth Self-Report (YSR), prior to psychiatric evaluation. Intake psychiatrists independently completed SWAP-AP ratings based on clinical evaluation, before reviewing self-report or parent report data. Each SWAP-AP prototype describes a personality configuration in “experience-near” language, with clinicians rating the match on a 1–5 scale (5=prototypical example).

**Results:**

The sample included 52% male participants, 35% Caucasian, 32% African American, 23% Hispanic, 3% Asian, and 7% “other”, with an average age of 17.77 years (SD=3.23). Forty-five SWAP-AP ratings were completed by clinicians, paired with available patient and parent data. The Emotionally Dysregulated prototype significantly correlated with: BYI scales of self-concept (r =−0.33, p=0.03), anxiety (r=0.36, p=0.01), and depression (r=0.50, p <0.001); IIP-32 self-sacrificing (r=0.42, p=0.03) and intrusive/needy (r=0.37, p =0.05) scales; YOQ social isolation (r=0.48, p=0.01) and depression/anxiety (r=0.41, p=0.03); and YSR anxious/depressed (r=0.48, p <0.01), withdrawn/depressed (r=0.43, p <0.01), and social problems (r=0.41, p =0.01).

**Conclusion:**

These results provide strong support for the construct validity of the SWAP-AP Emotionally Dysregulated prototype, which characterizes borderline personality pathology in adolescents. The observed correlations between clinician-rated SWAP-AP and patient self-reports align with theoretical expectations, indicating the prototype’s utility in capturing relevant psychopathology. Future research should investigate the predictive validity of the prototype matching approach and its sensitivity to treatment-related change.

